# The application of epidemiology in aquatic animal health -opportunities and challenges

**DOI:** 10.1186/1297-9716-42-94

**Published:** 2011-08-11

**Authors:** Edmund J Peeler, Nicholas GH Taylor

**Affiliations:** 1 Centre for Environment, Fisheries and Aquaculture Science (Cefas), Barrack Road, Weymouth, Dorset, DT4 8UB, UK

## Abstract

Over recent years the growth in aquaculture, accompanied by the emergence of new and transboundary diseases, has stimulated epidemiological studies of aquatic animal diseases. Great potential exists for both observational and theoretical approaches to investigate the processes driving emergence but, to date, compared to terrestrial systems, relatively few studies exist in aquatic animals. Research using risk methods has assessed routes of introduction of aquatic animal pathogens to facilitate safe trade (e.g. import risk analyses) and support biosecurity. Epidemiological studies of risk factors for disease in aquaculture (most notably Atlantic salmon farming) have effectively supported control measures. Methods developed for terrestrial livestock diseases (e.g. risk-based surveillance) could improve the capacity of aquatic animal surveillance systems to detect disease incursions and emergence. The study of disease in wild populations presents many challenges and the judicious use of theoretical models offers some solutions. Models, parameterised from observational studies of host pathogen interactions, have been used to extrapolate estimates of impacts on the individual to the population level. These have proved effective in estimating the likely impact of parasite infections on wild salmonid populations in Switzerland and Canada (where the importance of farmed salmon as a reservoir of infection was investigated). A lack of data is often the key constraint in the application of new approaches to surveillance and modelling. The need for epidemiological approaches to protect aquatic animal health will inevitably increase in the face of the combined challenges of climate change, increasing anthropogenic pressures, limited water sources and the growth in aquaculture.

**Table of contents:**

*1 *Introduction *4*

*2 *The development of aquatic epidemiology *7*

*3 *Transboundary and emerging diseases *9*

3.1 Import risk analysis (IRA) 10

3.2 Aquaculture and disease emergence 11

3.3 Climate change and disease emergence 13

3.4 Outbreak investigations 13

*4 *Surveillance and surveys *15*

4.1 Investigation of disease prevalence 15

4.2 Developments in surveillance methodology 16

4.2.1 Risk-based surveillance and scenario tree modelling 16

4.2.2 Spatial and temporal analysis 16

4.3 Test validation 17

*5 *Spread, establishment and impact of pathogens *18*

5.1 Identifying routes of spread 18

5.1.1 Ex-ante studies of disease spread 19

5.1.2 Ex-post observational studies 21

5.2 Identifying risk factors for disease establishment 23

5.3 Assessing impact at the population level 24

5.3.1 Recording mortality 24

5.3.2 Farm health and production records 26

5.3.3 Assessing the impact of disease in wild populations 27

*6 *Conclusions *31*

*7 *Competing interests *32*

*8 *Authors' contributions *32*

*9 *Acknowledgements *33*

*10 *References *33*

## 1 Introduction

Epidemiology was originally considered as the study of pathogen lifecycles, but is now more comprehensively defined in the Oxford English dictionary as "*the branch of medicine which deals with the incidence, distribution, and possible control of diseases and other factors relating to health*". The terms "epizootic" and "epizootiology" have been used in the veterinary literature but in keeping with current practice we refer to "epidemics" and "epidemiology" in this paper [1]. The birth of modern veterinary epidemiology can be traced back to work by Schwabe [2], who identified that traditional approaches to disease control were failing to resolve animal health problems occurring in intensive livestock production systems. From its inception, modern epidemiology was primarily an applied discipline, practised in the field, with the purpose of preventing and resolving animal health problems. The requirement for inter-disciplinary approaches was central to modern epidemiology, and it encompasses aspects of biostatistics, animal health economics, risk analysis and theoretical modelling studies [3]. A founding principle of modern epidemiology is that disease is caused by multiple interacting factors, which was formulated in the early 1970s as the causal triad of pathogen, host and environment [4]. However, it has since been argued that pathogens should be considered as a component of the environment [5].

Until relatively recently, epidemiological research focused on human and terrestrial animal systems with comparatively little effort directed towards aquatic animal health. However, with 71% of the Earth's surface being covered in water, we live on a wet planet. Life began in water, and the diversity of aquatic organisms is greater than on land. For example, viruses are the most abundant life form in the oceans. There are estimated to be millions of virus-like particles in every millilitre of seawater [6] and that every second 10^23 ^viral infections occur in the marine environment [7].

Humans use the aquatic environment for a range of services: potable water, wild capture fisheries, aquaculture, irrigation, travel, recreation and waste disposal. The interaction between humans and aquatic environment means that the study of pathogens in aquatic animals is important to protect a valuable food and recreational resource, and becaise diseases of humans may originate in the aquatic environment [8]. Globally aquaculture and fisheries supplied 110 million tonnes of food in 2006, of which 47% was accounted for by aquaculture [9], compared with only 3.9% in 1970 [10]. The majority of wild capture fisheries are over-exploited [9], growth in consumer demand and new technologies has led to a 10% per annum growth in aquaculture over between 1970 to 2008 [9]. Over 230 aquatic animal species are now farmed [10]. The development of aquaculture has, to a significant extent, depended on the use of non-native species [11] (e.g. marine culture of Altantic salmon in Chile and tilapia and sea-bass in land-based systems in the UK). The diversity and number of species being produced has also resulted in new culture systems and practices, which themselves could influence the emergence and spread of pathogens and occurrence of disease [12]. For example, polyculture (keeping several species on the same site) provides the opportunity for pathogen transmission between species [13]. Farmed fish may be exposed to wild aquatic animals and their pathogens, to which the they would not normally exposed. Similarly, aquaculture may expose wild fish to novel pathogens. Both cases are illustrated by tuna ranching in southern Australia. Juvenile tuna are caught in the open ocean and moved inshores, where they come in to contact with parasites to which they would normally have little or no exposure, resulting in disease emergence [14-16]. Additionally, the tuna are fed imported frozen fish which was very likely to have been the route of introduction of pilchard herpes virus, which has caused epidemics in wild populations [17].

Fish are one of the most commonly kept companion animals (in the UK there are estimate to be 135 million [18]) and as a consequence the global trade in ornamental aquatic animals is massive. Over 1 billion ornamental fish comprising more than 4000 freshwater and 1400 marine species are traded internationally each year [19,20]. Trade in live fish (for aquaculture, food and as ornamental animals) and changing climates has expanded the geographic range of many aquatic animal species (and their pathogens), facilitating disease emergence through host-switching [21-23].

**Figure 1:**
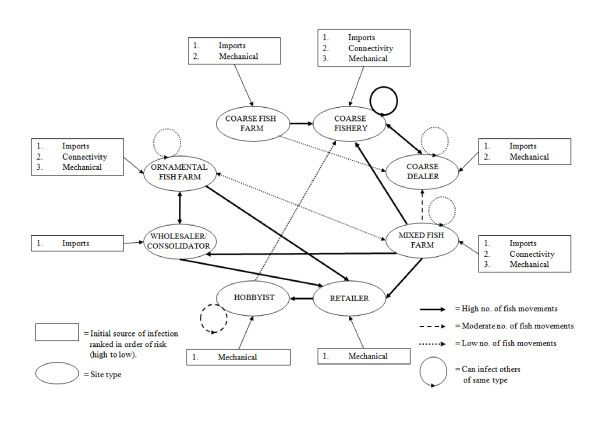
**Contact network structure occurring between businesses in the UK cyprinid fish sector, demonstrating the potential routes of pathogen introduction and spread (connectivity refers to sites being connected by the river network, and mechanical refers to transmission via vectors and fomites)**.

The uses of aquatic animals produce interactions between industry sectors that are not obvious and may drive disease emergence and pathogen spread. For example, within the UK cyprinid fish (e.g. carp and goldfish) industry there are two main interacting sectors: the ornamental and coarse (sport) fish (Figure 1). In summary, cyprinid fish may be imported from both European and non-European countries, the majority of which will be intended for the ornamental (fish keeping) sector. In addition cyprinid farm production with the UK supplies both sectors. Fish are also moved between fisheries either directly or via dealers. Data on fish movement in this network is patchy. It is believed that many movements between fisheries are non-consented and therefore not officially recorded [24], and additionally hobbyists are known to stock fish into lakes and rivers [19,25-27]. The cross-over that occurs between the ornamental and coarse fishing sectors has the potential to contribute substantially to pathogen spread in fisheries, and is believed to have been one of the main drivers in the early stages of the koi herpesvirus epidemic in the UK [24].

The diversity and interactions of aquatic systems presents a series of unique challenges and complexities that requires the holistic approach which is fundamental to modern epidemiology. This review aims to summarise the application of aquatic animal epidemiology in three key areas of aquatic animal health: i) transboundary and emerging aquatic animal diseases, ii) disease monitoring, surveillance and surveys, and iii) spread and impact of aquatic animal pathogens.

In addressing these questions, epidemiological approaches needed for diseases of livestock are compared with those required by studies of aquatic animal disease. Examples of where these approaches have been applied, their success and limitations, are discussed. Unique challenges and constraints that arise in epidemiological studies of aquatic systems are identified. The underlying theme running through this paper is the role of epidemiology in improving aquatic animal health.

## 2 The development of aquatic epidemiology

It has been argued that fish health research has focused on isolating and characterising pathogens, at the expense of host and environmental factors [28]. Only relatively recently have epidemiological studies investigated causes of aquatic animal diseases under field conditions. Inevitably the development of epidemiological methodology has focused firstly human, and secondly terrestrial livestock health issues. As a consequence the development of aquatic epidemiology has lagged behind its terrestrial counterparts, however in recent years the field has developed rapidly. Epidemiological studies of aquatic animals systems are becoming more common place and the techniques used have increased in complexity.

The beginning of aquatic animal epidemiology mirrored the evolution of modern epidemiology in terrestrial livestock. The first risk factor study was published by Thorburn [29], who applied approaches previously used in terrestrial systems to investigate mortality due to vibriosis in farmed rainbow trout. Intensive Atlantic salmon (the first large scale commercially produced fish) production was pioneered in Norway in the 1970s. Inevitably, disease problems emerged, most notably infectious salmon anaemia (ISA) [30]. ISA and other diseases stimulated epidemiological research to support cost-effective control strategies in farmed Atlantic salmon [31-33]. Subsequently, other important diseases have emerged in aquaculture, some with serious economic and environmental consequences. *Gyrodacytlus salaris *emerged in Norway in the early 1970s causing serious declines in many wild Atlantic salmon populations [34]. Farmed Atlantic salmon may act as a reservoir for infection, thus contributing to populations declines in wild salmon [35]. A number of viral diseases, notably white spot syndrome virus (WSSV), have emerged in the nascent paenid shrimp farming sector, with devastating economic consequences in some countries [36]. Cold water strawberry disease emerged in 2006 in the UK, causing financial losses to trout producers [37]. Disease emergence has clearly provided an important spur to the application of epidemiology.

The growth of aquatic animal epidemiology is illustrated by an analysis of the number and type of studies presented at the International Society of Veterinary Epidemiology and Economics (ISVEE) conferences, held every three years since 1976 (Table 1). The number of aquatic abstracts and the species being studied have increased, the questions addressed and the techniques used have become more diverse (the location of the conference may influence the number of aquatic animal submissions). Shellfish (molluscs and crustacea) production accounts for over a quarter of the aquatic animals consumed annually [9], but have been the subject of comparatively few studies (which are predominantly of cultured shrimp). This may be explained by the limited options for disease control.

**Table 1:** Analysis of oral presentations at meetings of the International Society for Veterinary Epidemiology and Economics (ISVEE), 1976-2009

Year	Total papers presented	Aquatic papers presented	Species (number of presentations)	Subject area (number of presentations)
1976 - 1982	246	0 (0%)		

1985	117	1 (0.85%)	Fish (1)	Economics/Control (1)

1988	164	1 (0.61%)	Fish (1)	Diagnostic tools (1)

1991	207	2 (0.97%)	Fish (2)	Control (1) Pathogen dynamics (1)

1994	228	0 (0%)	NA	NA

1997	423	9 (1.83%)	Fish (7) Shellfish (2)	Baseline/monitoring/investigation (6) Control (1) Risk analysis (1) Risk factors (1)

2000	603	15 (2.49%)	Fish (13) Shellfish (2)	Baseline/monitoring/investigation (6) Risk factors (4) Control (2) Diagnostic testing x2 Risk analysis (1)

2003	691	19 (2.75%)	Fish (16) Shellfish (3)	Baseline/monitoring/investigation (7) Risk factors (4) Risk analysis (2) Zoonosis (2) Epidemiological tools (2) Disease modelling (1) Control (1)

2006	911	67 (7.35%)	Fish (41) Shellfish (12) Amphibians (4) General (10)	Baseline/monitoring/investigation (25) Risk factors (13) Epidemiological tools (9 Control (6) Risk analysis (5) Diagnostic testing (4) Pathogen dynamics (2) Disease modelling (1) Zoonosis (1) Economic (1)

2009	755	26 (3.44%)	Fish (24) Shellfish (1) General (1)	Baseline/monitoring/investigation (11) Risk factors (6) Epidemiological tools (2) Control (2) Risk analysis (2) Diagnostic testing (1) Pathogen dynamics (1) Disease modelling (1)

## 3 Transboundary and emerging diseases

A key role for epidemiology is to identify and assess exotic and emerging disease threats. Transboundary diseases are a major threat to both farmed and wild terrestrial and aquatic animal populations [21]. In recent years, a number of aquatic animal diseases have emerged and spread rapidly across international political boundaries, causing serious financial and ecological impacts. WSSV emerged in shrimp (*Paenus monodon*) culture in China (Fuijan Province) in 1992 and subsequently spread to Taiwan and Japan and then became panzootic [36]. The main route of transmission was the movement of live animals [36]. *G. salaris *emerged in Norway in the early 1970s, and is hypothesised to have been introduced from Sweden with the introduction of a Baltic strain of Atlantic salmon [34]. A new variant of oyster herpes virus recently emerged in European oyster production [38] and has spread rapidly through live animals movements. Koi herpesvirus achieved widespread distribution across the globe within a few years of emerging through movements of carp, mainly destined for the ornamental trade industry [39]. The movements of bullfrogs (as pets, food and for laboratory use) has been implicated in the spread of the fungal disease, chytridiomycosis in wild amphibia [40]. Movements of animals drive disease emergence not only through extending the geographic range of known diseases but by moving species outside of their natural ranges, thus providing the opportunity for putative pathogens to find new hosts [22]. The scale and diversity of aquatic animal movements, mainly for the ornamental trade but also for food and aquaculture, far outweighs international terrestrial movements, and presents important challenges to epidemiologists.

### 3.1 Import risk analysis (IRA)

IRA assesses the risk of disease spread through trade in animals or their products. In aquatic animal health they have been undertaken for a variety of reasons (reviewed by Peeler et al., [41]), but mainly to support animal health policy making with respect to trade and biosecurity. Epidemiological approaches are used within the import risk analysis framework [42]. Consistent, defensible and scientifically sound assessments of disease threats and disease freedom are required to fulfil the requirement of the Agreement on the Application of Sanitary and Phytosanitary (the SPS agreement) of the World Trade Organisation (WTO) [43] (which was enacted to allow Members to adopt measures to protect human, animal or plant life or health, whilst preventing restrictions on international trade disguised as sanitary measures). Thus this application of epidemiology, for both terrestrial and aquatic animal diseases, have developed in response to both requirements of international trade agreements and the real threat of transboundary diseases. One of first aquatic animal IRA was Biosecurity Australia's assessment of the risk of disease introduction with the import of wild caught salmon carcasses from Canada [44]. IRA requires information on the epidemiology of the diseases in the exporting country, biophysical characteristics of the pathogen and product information to be integrated. As part of the consequence assessment epidemiological and economic modelling are needed to assess disease spread and impact on local industries and wild populations. More broadly, an assessment of routes of introduction (through trade, movement of shipping, lorries etc.) is the necessary basis on which to review a country's biosecurity measures. However, there are constraints to IRA, as currently practised. Frequently, relevant information is not available. IRA cannot account for unidentified hazards; this is particularly relevant for aquatic animal health in general [45] and the trade in ornamental aquatic animals in particular [19].

### 3.2 Aquaculture and disease emergence

The same risk framework which has been used for examining disease spread through trade in live animals and animal products have also been applied to disease emergence. Murray and Peeler [12] and Bridges et al. [46] used risk models to investigate drivers for the emergence of disease in aquaculture. However, disease emergence in terrestrial systems has been studied in greater depth [47-49], in particular the evolution of virulence [50-52], the importance of animal reservoirs [53] and multiple hosts [54-56]. These studies have in large part been driven by the emergence of zoonotic pathogens [57,58]. Epidemiological studies need to more fully explore the conditions under which putative pathogens spread between wild and farmed aquatic animals. The introduction of non-native species has been fundamental to the expansion of aquaculture. Rainbow trout, a native of North America, is the most important freshwater finfish species in Europe and is cultured across the globe [59]. *Paenus monodon *and other species have been shipped internationally to establish shrimp aquaculture. A recent review demonstrated that the introduction of non-native aquatic animal species underpinned many instances of disease emergence in Europe [22] and this is likely to be true for other parts of the world. It is likely that the culture of new species, the expansion of aquaculture into new areas and the global trade in both live aquatic animals and commodity will continue to increase as the demand for animal protein increases with the growth in human population and the decline of wild capture fisheries [10]. Diseases which emerge in farmed populations invariably originate in wild populations. Thus interaction between wild and farmed populations facilitated by the open design of many aquaculture systems [60,61] and the current fish farming practices [62] is fundamental to disease emergence. Additionally, restrictions in the use of chemicals or the development of resistance could lead to the re-emergence of old diseases that had previously be controlled. Applying the epidemiological modelling approaches which have been used to study disease emergence in terrestrial systems may provide insights into the processes driving disease emergence in the aquatic environment, and inform the development of improved mitigation measures.

### 3.3 Climate change and disease emergence

Climate change will influence the development of aquaculture and the epidemiology of aquatic animal diseases. Many important aquatic animal pathogens have temperature thresholds, above or below which disease or infection does not occur. A risk assessment model to screen pathogens, to identify those whose threat will increase under established climate change scenarios has been developed for freshwater fish diseases and applied to the UK [23], which identified *Lactococcus garvieae *and *Anphanomyces invadans *as the exotic diseases whose threat will increase most with rising water temperatures. Epidemiological modelling and the application of geographic information systems (GIS) have been used to good effect to assess the likely impact of climate change scenarios on terrestrial livestock diseases (especially vector borne diseases such as blue tongue virus and trypanosomiasis) [63], and huge scope exists to apply these approaches to aquatic animal diseases.

### 3.4 Outbreak investigations

Outbreak investigation is a core activity for epidemiologists and uses key methods of modern epidemiology: measurement of disease frequency in time and space and in different populations. More recently molecular analyses have been incorporated into outbreak investigations in order to aid source tracking. Outbreak investigations of transboundary diseases are generally undertaken by the veterinary services and generally remain unpublished. However, research papers investigating the origin of transboundary diseases have been published. Investigations of infectious haematopoietic necrosis virus (IHNV) in Europe have attributed its introduction to imported rainbow trout eggs from the US [64]. This hypothesis has been supported by molecular epidemiology studies showing that all European IHNV isolates belong to one of the four N. American genogroups [65]. Conversely, genotyping demonstrated that viral haemorrhagic septicaemia virus (VHSV) in N. America strain had emerged separately from a marine reservoir with no epidemiological link to Europe [66,67] (where the disease first emerged). Similarly genotyping of ISAV demonstrated emergence from the marine reservoir on separate occasions in Norway, Scotland and N. America [68,69]. However, genetic analysis of the isolate from the recent outbreak in Chile is identical to Norwegian isolates suggesting spread via imported eggs in 1996 [70,71].

Despite its growing use and popularity, aquatic molecular epidemiology is yet to reach the level of sophistication applied to terrestrial systems. Examples of the potential power of molecular epidemiology can be seen in studies such as Cottam et al. [72] who, in studying the transmission of foot and mouth disease virus (FMDV) used small mutations found in the entire viral sequence to identify possible previously unidentified contacts in the 2001 epidemic. The availability of technologies that allow for rapid, large-scale sequencing, such techniques provide exciting potential to develop aquatic epidemiology, and may provide a powerful tool to enhance our ability to accurately trace the source and course of infections.

## 4 Surveillance and surveys

### 4.1 Investigation of disease prevalence

Epidemiological textbooks [73,74] generally define disease monitoring as ongoing efforts to assess health and disease in a specified population. The term surveillance is reserved for a more active system and implies action will be taken based on the results. Surveys collect information for a specific aim or to test a hypothesis. Monitoring and surveillance for aquatic animal disease is undertaken by governments to demonstrate disease freedom or progress in disease control, and generally remains unpublished (the exception being work by East et al. [75] demonstrating freedom in Australia from WSSV). In the scientific literature there are a number of reports of surveys of disease in farmed and wild fish. Studies of gyrodactylid infections in farmed rainbow trout in Denmark [76,77] showed considerable between farm and seasonal variation. Investigations of the prevalence of renal myxosporidiosis in wild [78-81] and farmed salmonids [82] were undertaken to assess the impact of these diseases. Studies of *Renibacterium salmoninarum *(Rs) revealed a low prevalence in wild salmonid fish populations [83] and farms [84], and considerable variation between rivers and farms. More recent a sero-survey of koi herpesvirus found that the virus was geographically widespread in fisheries in E&W, but largely absent from farms [25].

Relatively few papers have used more sophisticated spatial and statistical analytical methods However, analysis of data from a large-scale survey of Atlantic salmon in Scotland [85]) modelled the within and between river prevalences to correct the bias that arose from low sample sizes (in some rivers) and pooling [86]. Peeler et al. [81] used multi-level modelling approaches to analyse prevalence data of renal myxosporidiosis and hepatitis in wild brown trout, and concluded that site (compared with factors at the level of the fish or river) level factors exerted most influence.

### 4.2 Developments in surveillance methodology

In terrestrial animal epidemiology there has been considerable progress in the development of methods and approaches to support the design and analysis of surveillance systems and data [73]. In this section, the application of these methods to aquatic animal diseases is discussed.

#### 4.2.1 Risk-based surveillance and scenario tree modelling

Risk-based surveillance (RBS) and scenario tree modelling are powerful tool for improving the design and analysis of surveillance systems, not least because complex data sources can be used and historic data discounted [87,88]. To date there are only publications on the use of RBS and scenario tree modelling in terrestrial systems, however, these method have started to be used in aquatic animal production systems. The more sophisticated approaches used in terrestrial animal surveillance have not yet been applied to aquatic systems, in part, because of an inevitable lag that will occur for methods developed for one system to be refined and used elsewhere. The EU aquatic animal health directive 2006/88/EC requires member states to use RBS, and so will drive the application of this approach in aquatic systems.

#### 4.2.2 Spatial and temporal analysis

The use of for example the scan statistic [89] to detect spatial and temporal clusters or the nearest neighbour technique (first developed by Clark and Evans [90]) to detect spatial clusters, helps identify confounding and risk factors for disease, and generate research hypotheses [91,92]. These techniques could serve the same role in aquatic animal health research, though spatial analysis would need to account for hydrodynamic connections between populations (e.g. for farms in the same river catchment). In the terrestrial field, data from long term data collection exercises have been used. These have often been collected for statutory purposes (e.g. monitoring for bovine spongiform encephalopathy). Arguably, it is the lack of suitable datasets which have held back the adoption of these approaches. The main exception to this are datasets of sealice infections of farmed Atlantic salmon [93-95] and a single dataset of 14 years of monitoring of two cyprinid populations and their parasites in three rivers in north east England [96], which could be used to assess parasite burdens over time.

### 4.3 Test validation

Advances in Bayesian statistical methods to determine test characteristics (sensitivity and specificity) in the absence of a gold standard (TAGS) have also contributed to the development of surveillance [97,98], and are of great importance in aquatic systems as few true gold standard methods currently exist. However, the test characteristics of relatively few diagnostic tests have been validated under field conditions and in the absence of a gold standard, the exception being tests for infectious salmon anaemia [99,100]. The techniques and expertise exists; once again the main constraint is the lack of resources to test a sufficient number of populations (with different levels of disease). Pooling diagnostic samples occurs more frequently with aquatic compared with terrestrial animals, with many of the tests approved by the World Organisation for Animal health [101] for aquatic animal diseases allow samples from up to 10 animals to be pooled. The effect of pooling on the characteristics of these test have in general not been considered, however, methods to adjust prevalence estimates for pooled samples have been developed [102]. Epidemiologists have an important role to play in designing trials to generate information on test characteristics and to assess the impact of pooling [97].

## 5 Spread, establishment and impact of pathogens

### 5.1 Identifying routes of spread

Understanding the routes of disease spread is of key importance to containment and control. Transmission mechanisms must first be identified and understood, and then the importance of routes of transmission between areas or sites quantified. Studies of the UK 2001 foot and mouth disease (FMD) epidemic provide examples of techniques which may be of use in the study of aquatic systems: farmed based stochastic modelling [103]; social network analysis [104] and space time interaction [105]. As in terrestrial systems, the main routes of transmission in aquatic systems are the movement of live animals, transmission via vectors and fomites and the contiguous nature of some sites (by connecting water sources). The specific processes involved for each route and their importance can be investigated ex-ante by theoretical probabilistic approaches (e.g. risk assessment), and ex-post observational studies of patterns of disease spread.

#### 5.1.1 *Ex-ante* studies of disease spread

Peeler et al. [41] reviewed the use of risk assessment to study the spread of aquatic animal pathogens between countries, river catchments and farms. A number of risk assessments focused on *G. salaris*. These ex-ante studies tended to focus on one pathway and addressed specific management questions. Paisley et al. [106] used Monte-Carlo simulation methods to study the likelihood of *G. salaris *being introduced to the river Tana in Norway from infected Atlantic salmon smolts in marine cages. The study identified the components of this route that had the most influence in determining the final probability of introduction (salinity at the cage site followed by the time of year fish escaped from the farm). Høgasen et al. [107] used similar methods to assess the risk associated with infected wild Atlantic salmon smolts moving to uninfected catchments and successfully transmitting *G. salaris*. Peeler, Gardiner and Thrush [108] conducted a qualitative risk assessment to establish the most important routes by which *G. salaris *would spread through England and Wales should it be introduced. This study concluded that in England and Wales the most likely transmission routes were the movement of live rainbow trout followed by the movement of other fish species. Such studies provide valuable information for contingency plans to prevent spread should an introduction occur.

Once the contact pathways have been established, their importance in an epidemic and the overall rate of spread can be modelled between sites (or individuals as is often the case with human pathogens). This however relies on an accurate picture of the total population size of susceptible, potential reservoirs, and the degree of contact between them. If the question is simply to determine the rate of pathogen spread under different conditions, simple compartment based epidemic models, in which the host moves from susceptible to infected states [109], may be applied. Murray [110] and Ruane et al. [111] used such models to study the spread and dynamics of infectious pancreatic necrosis virus (IPNV) between salmon farms in Scotland and Ireland, respectively. Both studies suggested that the stocking of infected smolts from freshwater sites drove the epidemic in the marine sites, but Ruane et al. [111] found that endogenous spread between marine sites took over as the dominant transmission route shortly after introduction from freshwater sites. Taylor et al. [112] also found that the role of infection pressures in spreading a pathogen could change as an epidemic progressed: the stocking of imported ornamental fish drove the initial phase of the koi herpesvirus epidemic in England and Wales (E&W), but that live fish movements between lakes became the main driving force after a few years.

Network analysis may be employed to investigate which sites are most likely to be exposed to pathogens. The technique is used to investigate the number and direction of contacts (edges) made by one site to another, and allows several useful measures that may facilitate an understanding of pathogen spread to be derived. In their simplest form they simply model contacts and not transmission. However, they can be combined with epidemic models, and simulations run to determine how a disease might spread through a population. Such approaches are powerful, but rely on a full knowledge of the network structure, which is also assumed to be stable over time, i.e. sites always contact the same sites. In aquatic systems, network approaches have received relatively little attention, as the required contact data is often not available. However, some systems do lend themselves to this approach, and two good examples of this are demonstrated by Thrush and Peeler [113], who looked at live trout movements in E&W, and Green [114] who studied live fish movements between Scottish trout and salmon sites. In the UK, data on live fish movements from farms exists farmers are obliged to keep these records. Both studies used these data to construct contact networks. Thrush and Peeler developed a stochastic model to examine how rapidly a pathogen may spread on the network. Green et al. [115] used a network model to identify sites where movement controls should be targeted to most effectively slow the spread of disease. Sharkey et al. [116] further developed the work of Thrush and Peeler [113] by adding other pathways such as connectivity via the river network to the analysis. Hydrographical connections between farm sites have also been studied in the marine environment using particle tracking, advection decay models. These have been used to good effect in the study of the dispersal of sea lice [117], ISAV [118], pancreas disease virus [119] and IPNV [120]. If network and hyodrographical models were accurately parameterised, for a particular pathogen, they could be used during an outbreak to estimate the likely spread of the pathogen. In addition they can be used to develop outbreak scenarios which to test contingency plans and specifically competing control measures. Such studies are, however, often limited by a lack of available data on the contact network and key epidemiological parameters such as transmission rate, latent periods, duration of shedding and immunity.

#### 5.1.2 *Ex-post* observational studies

Retrospective observational studies (e.g. case control studies) of spread are a natural extension of outbreak investigations. They have been used extensively in terrestrial epidemiology to identify routes of spread, e.g. analysis of data from the FMD 2001 outbreak [121]. Again *G. salaris *provides an example of a case-control study [122] which used logistic regression to assess routes of spread (whilst accounting for confounding variables); and concluded that the amount of freshwater inflow into fjords and the distance travelled by infected fish were the most important factors. A number of retrospective studies of ISA have provided important insights into routes of spread. Early work in Norway demonstrated that spread from neighbouring infected farms and slaughter houses were important [32,123]. Mechanical routes of transmission (e.g. divers moving between sites) were also significant [123]. Investigations of the ISA outbreak in Scotland [124] and Canada [125] identified the use of well boats, for moving and harvesting fish, as an important factor in the spread of the disease.

The limitation of studies such as case-control are that it is often not known if the pathogen has been introduced to a control site but has not be detected as conditions at the site were not conducive to disease expression or establishment. Serological testing may provide insight into whether this may have been the case, and was used by Taylor et al. [24] to identify carp fishery sites that may have been exposed to koi herpesvirus. Subsequent contact tracing studies also using such tests subsequently suggested that live fish movements were the main route of transmission [126]. Unfortunately, at present few validated serological test methods exist for aquatic animal diseases.

### 5.2 Identifying risk factors for disease establishment

Classical epidemiological studies such as cross-sectional, case-control and cohort are commonly applied to identify risk factors associated with pathogen establishment and disease. In terrestrial systems individual animals were often the unit of study. In aquatic systems, although these study types are now commonly used by aquatic epidemiologists, they tend to be conducted at the level of the site (i.e. farm or fishery) or batch/cohort of fish due to the logistics of tracking individual fish.

There are a growing number of observational epidemiological studies that have identified risk factors for aquatic animal diseases. Most commonly these studies have undertaken to support the development of management advice; examples include diseases of farmed salmon [31-33], including sealice [127], and white spot syndrome virus (WSSV) infection in paenid shrimp [128]. The same approaches have also been applied to investigate risk factors for non-infectious causes of disease, for example fin erosion in rainbow trout [129], cataracts [130] and skin lesions [131] in Atlantic salmon. To date, few studies have focused on ornamental fish or fishery populations. With some exceptions these studies have investigated diseases in managed stillwater fisheries (e.g. *Argulus *spp. [132,133] and KHV [25]). Most of these studies use standard generalised linear modelling techniques such as logistic regression to determine risk factors, but due to the difficulties in accurately measuring many factors of interest, latent variable modelling techniques may provide an approach that should be considered in future studies. The lack of studies in ornamental fish and fishery systems is likely a reflection of difficulties in collecting data from these systems. However, with a growing focus on conservation of wild stocks, their study and interactions with other sectors is likely to be of increased interest and should be the focus of future research efforts.

Most of the risk factor studies have been cross-sectional, which have the advantage over case-control studies that they provide an estimate of disease prevalence, but are not suitable for studying factors which change over time. Few prospective longitudinal studies of disease frequency in aquatic animals have been published, possibly because these studies face a number of constraints not encountered in terrestrial systems. Animals are not individually identified, and on farms groups of fish are split and mixed during the production cycle. These factors make repeated observations over time problematic (possible solutions were discussed by Thorburn et al. [134]). However, successful prospective studies of WSSV in shrimp [128] and a cohort studies in Atlantic salmon [131,135,136] have been completed.

### 5.3 Assessing impact at the population level

It is important to have accurate assessments of the impact of disease at the population level to justify and prioritise expenditure on aquatic animal health. However, a range of impacts need to be considered. Most classically one thinks of mortality and the economic impact attributed to it; however there may also be indirect financial impacts. The key question about the impact of pathogens on wild populations is whether mortality, reduced fecundity, recruitment etc. actually reduce the overall population size, or whether their influence is mitigated by other density-dependent processes, i.e. can impacts at the level of the individual be scaled to the population.

#### 5.3.1 Recording mortality

Although mortality is a commonly used measure of impact, in the aquatic environment even this may not be easy to measure. In the fish farm environment dead and moribund fish are relatively easy to observe compared to wild fish or fishery populations, and there is generally a legal obligation on farmers to record mortalities. However due to the large number of fish that may die (especially in the case of juvenile fish), accurate recording may be a problem. Additionally many farms experience relatively high and variable levels of "natural" background mortality compared to terrestrial farms, and multiple infections may occur at the same time. Consequently, attributing a cause of death to each individual and identifying when a disease first occurs is not always easy (although with a time-series of mortality data simple statistical techniques such as cumulative sum plots are useful). Fish farmers will often record the most obvious potential cause (often an ectoparasite, as they are easy to see and identify), to which all mortalities are attributed. The quality and value of diagnoses by farmers is likely to be highly variable, and this must be taken into consideration when using it for the purpose of epidemiological studies.

Assessing mortality attributed to a disease in wild or even recreational fisheries is more difficult, and as a consequence has been little studied. Even small recreational fisheries are relatively extensive and deep compared to farm systems. The likelihood of detecting mortality or capturing moribund fish for the purpose of diagnosis is therefore low, especially in the early stages of and epidemic. Methods for assessing impact wild populations is discussed in section 5.3.3.

#### 5.3.2 Farm health and production records

Some farmers use production software that records mortalities and their causes. Such information is potentially of great use to the epidemiologist, but commercial sensitivities may constrain its use. Wheatley et al. [137] used production records from Irish salmon production to assess the impact of endemic diseases. Attempts have been made to create a national database to store production and mortality information for the UK trout industry (Development of a scheme for monitoring sentinel farms in the UK trout industry, Scottish Aquaculture Research Forum, project number 028). This database was designed to compile a national picture that would allow farmers to compare their sites performance with their sectors baseline performance (a common practice in cattle production where extensive health and production databases exist). Currently this system is of limited use to the research scientist, but a similar database, MonAqua which holds Norwegian salmon production data has successfully been used by epidemiologists to assess health related impacts [138]. The Faroe Islands have also successfully implemented such a database created following outbreaks of infectious salmon anaemia between 2000 and 2005, which could be used for similar purposes. This is a government initiative which requires by law that farmers report production figures, fish movements, mortalities and their causes on a regular basis for each cage. Where such systems are available, care must be taken to note the purpose of the database when compiling and analysing data, as data collected by the industry for their own monitoring purposes is likely to be subject to a different set of biases to that collected to comply with government regulations.

Of the few databases used to compile disease information in aquatic systems the most extensive are of sea lice infections in farmed Atlantic salmon. This is possibly due to the serious impact of this pathogen across the industry, and the fact that the majority of farms are owned by only a few companies that want to tightly monitor their lossess and the efficacy of treatments across sites. These data have been extensively used to build statistical and mathematical models of the epidemiology of *Lepeophtheirius salmonis *[139], *Caligus elongatus *[140] and other sea lice [141]. The studies include the identification of risk factors [127], time series analysis for trends [142] the impact of treatment timing [143], and economic impacts [144].

#### 5.3.3 Assessing the impact of disease in wild populations

Although the incidence and dynamics of disease in wild aquatic animal populations has been studied, relatively few have adequately assessed impact at the population level. A few observational studies have provided assessments of pathogen induced. Johnsen and Jensen [145] compared Atlantic salmon catch rates and parr densities in Norwegian rivers where *G. salaris *was known to be present and absent. Although no formal statistical associations were provided, the data clearly demonstrated a decline in infected compared with uninfected salmon populations. Several longitudinal studies have been conducted to establish statistical associations between pathogen prevalence/abundance and impact. Taylor et al. [146] for example, studied the impact of *Argulus foliaceus *abundance and other risk factors on fish capture rates in several recreational trout fisheries and demonstrated a statistically significant reduction in capture rates when the parasite burden was high but it was not possible to quantify host mortality. Murray et al. [147] used mark recapture methods combined with non-lethal pathogen sampling methods in a longitudinal study to assess the impact of chytridiomycosis, demonstrating a clear decline in frog survival attributable to the infection.

The effort required to collect the data sets required to conduct observational studies is costly and labour intensive, especially if the host under study is not of interest in terms of commercial fishing, and a dedicated survey is therefore needed. Due to their extensive nature, such surveys are difficult in freshwater systems, but exceptionally demanding in marine systems. Consequently, theoretical modelling approaches that specifically model the host-pathogen interaction may be more appropriate (given knowledge of host-pathogen life cycles and processes), since they provide a method of extrapolating data on individual host level impacts to the population level. The application of such techniques is reflected in the scientific literature. The majority of these modelling studies are based on deterministic differential equation based models, but the model structure can vary greatly in nature. Patterson [148] extended a fisheries capture model to incorporate different mortality rates for infected and uninfected fish. This was used to study the influence of *Ichthophonus hoferi *infections in herring and demonstrated a 21% reduction in stock size. The study highlighted one of the short comings of standard fisheries models, in that they normally do not account for dynamic natural mortality processes such as disease.

The impact of pathogens reproducing on fish farms and effecting wild stocks is a major area of concern, and a promising subject for theoretical studies. Krkošek et al. [35] modelled the impact of sea lice (*L. salmonis*) emerging from Canadian fish farms on the survival of two species of migrating wild Pacific salmon. Deterministic differential equation based models were combined with several different model types. They used an advection-diffusion-decay model to track parasite dispersal and transmission, and coupled this to a survival model for the two host species. The study showed that the sea lice substantially reduced host survival, and that fish farms produced the number of parasite required to induce large scale population effects.

Other approaches to modelling have also been used to good effect in studying the impact of pathogens in wild aquatic animal populations. Borsuk et al. [149] used a Bayesian probabilistic network to study declining brown trout populations in Swiss rivers. This network incorporated many factors thought to be influential in the life-cycle of this fish. The parasite *T. bryosalmonae *(the aetiological agent of proliferative kidney disease) was one of the factors included, and the analysis demonstrated that this was one of the most influential factors in driving population declines, especially at higher temperatures. The approach provides evidence to support recommendations for risk mitigation and the identification of sites that are likely to be at high risk, thus allowing monitoring efforts to be targeted.

One of the major challenges in modelling the impact of epidemics and their impact in wild aquatic animal populations is they can occur over vast areas in many distinct populations and subpopulations. Murray and O'Callagan [150] addressed some of these issues in their review of the different approaches used to study the pilchard herpesvirus epidemic in Australian stocks in 1998 to 1999. The authors demonstrated the need to apply several different model types to understand the processes occurring at different scales; from the epidemic within a shoal, to between shoal spread, and the large scale transmission around the southern Australian coastline.

In summary, epidemiological studies to study pathogen impact in wild aquatic animal populations are sparse compared to farmed species. This is in part likely to be due to the challenges of data collection, the scales over which these processes occur, and the many interacting factors. Adjusting for the effect of density-dependent processes may pose a significant challenge in accurately assessing impact, however, in exploited populations such as wild capture fisheries it may be argued that such processes are of less concern. Sub-lethal effects of pathogens (such as reduced growth or fecundity) that are often observed at the individual level are also difficult to assess at the population level, and modelling approaches provide and important tool in their assessment.

A lack of research into the impact of pathogens on wild fish populations may be because it is difficult to influence events, and some fisheries scientists may argue that pathogen-induced mortality is of little consequence in the presence of high fishing mortality. However, with increasing pressures on fish stocks, understanding the influence of these combined pressures on a population may be of great importance when trying to identify tipping points and set catch limits, in order to prevent population collapses. Many of the challenges described here for the study of impacts in fisheries and wild aquatic animals are similar to those faced by researchers studying wildlife epidemiology and population ecology. These disciples may provide further tools helpful to the study of aquatic systems (e.g research on the impact of parasites on gamebirds [120,151] and rabies in wildlife populations [20]).

## 6 Conclusions

Georgiadis et al. [152] reviewed how epidemiology was and could be used in aquatic animal health. Peeler et al. [41] reviewed the application of risk analysis in aquatic animal health management. In this review we have tried to demonstrate the wide breadth of epidemiological studies and the range of tools, approaches currently being applied to study disease aquatic systems, and the constraints and challenges facing epidemiologists. The single unifying actor is the purpose of epidemiology, namely the improvement of animal health. Although epidemiological studies in aquatic systems have, and continue to lag behind those applied to terrestrial systems in terms of scope and complexity, rapid development in the application of epidemiology to aquatic systems over a relatively short period has occurred. Consequently, epidemiological studies have made a significant contribution to the health of both wild and farmed aquatic animals, through improved biosecurity and surveillance of exotic diseases and control of endemic diseases.

The role for epidemiology in aquatic animal health became clear as pathogens emerged and rapidly spread in intensive aquaculture systems and disease interactions between wild and farmed populations became increasingly problematic. These complex aquatic animal health issues required inter-disciplinary approaches and epidemiologists are best placed to integrate and analyse diverse data sources and lead teams of pathogens specialists, economists, modellers and statisticians. Despite its success to date many challenges remain. Difficulties in obtaining data on host populations, especially in extensive systems require epidemiologists to work with fishery scientists and ecologists to better integrate wild fish demographics into epidemiological studies and models. Economic, population and disease model needed to be better integrated to improve assessments of disease spread to support aquatic animal health policy making. Surveillance methods developed for terrestrial systems need to be adapted for aquatic animals, and the lack of diagnostic test methods suited to surveillance (as opposed to disease confirmation) is currently a major limitation. More sophisticated models of the processes underlying disease emergence that take into account, *inter-alia*, the role of multi-host pathogens and reservoir populations and a greater understanding of the way pathogens are transmitted in aquatic systems are needed to support measures to minimise the number and impact of new diseases.

The volume of both legal and illegal trade in live aquatic animals far surpasses that of terrestrial animals, and is likely to be the biggest challenge in trying to prevent the spread of emerging aquatic diseases. Additionally, over the next few decades, not only is such trade set to increase, but aquaculture is likely to further grow, and pressures of limited water resources and climate change effects are likely to combine to increase the rate at which diseases emerge and spread. In the face of these combined challenges the demand for epidemiological approaches to support measures to protect aquatic animal health will undoubtedly increase.

## 7 Competing interests

The authors declare that they have no competing interests.

## 8 Authors' contributions

The authors contributed equally to this review. Both authors read and approved the final manuscript.

## 9 Acknowledgements

This work was supported by research funding (FC 1185 and FB001) from the Department for Environment, Food and Rural Affairs (Defra, UK).

## References

[B1] Dohoo IR Morris RS Martin SW Perry BD Bernardo T Erb H Thrusfleld M Smith R Welte VR Epidemiology Nature 1994 368 284 8127358 10.1038/368284c0

[B2] Schwabe C The current epidemiological revolution in veterinary medicine. Part 1 Prev Vet Med 1982 1 5 15 10.1016/0167-5877(82)90003-4

[B3] Thrusfield M Veterinary Epidemiology 2005 3 Blackwell Science Ltd

[B4] MacHohan B Pugh TF Epidemiology: Principles and Methods 1970 Boston: Little Brown

[B5] Martin SW Meek AH Willeberg P Veterinary Epidemiology - principles and methods 1987 1 Iowa: Iowa Statue University Press/Ames

[B6] Bergh O Borsheim KY Bratbak G Heldal M High abundance of viruses found in aquatic environments Nature 1989 340 467 468 10.1038/340467a0 2755508

[B7] Suttle CA Marine viruses [mdash] major players in the global ecosystem Nat Rev Micro 2007 5 801 812 10.1038/nrmicro1750 17853907

[B8] Taylor NGH Verner-Jeffreys DW Baker-Austin C Aquatic systems: maintaining, mixing and mobilising antimicrobial resistance? Trends Ecol Evol 2011 26 278 284 10.1016/j.tree.2011.03.004 21458879

[B9] F.A.O State of the World Fisheries and Aquaculture Book State of the World Fisheries and Aquaculture 2008 City: Food and Agriculture Organisation Fisheries and Aquaculture Department 196

[B10] Subasinghe RP Epidemiological approach to aquatic animal health management: opportunities and challenges for developing countries to increase aquatic production through aquaculture Prev Vet Med 2005 67 117 124 10.1016/j.prevetmed.2004.11.004 15737426

[B11] Gozlan RE Introduction of non-native freshwater fish: is it all bad? Fish Fish 2008 9 106 115

[B12] Murray AG Peeler EJ A framework for understanding the potential for emerging diseases in aquaculture Prev Vet Med 2005 67 223 235 10.1016/j.prevetmed.2004.10.012 15737433

[B13] McInerney J Old economics for new problems - livestock disease: presidential address J Agric Econ 1996 47 295 314 10.1111/j.1477-9552.1996.tb00695.x

[B14] Aiken HM Hayward CJ Nowak BF An epizootic and its decline of a blood fluke, Cardicola forsteri, in farmed southern bluefin tuna, Thunnus maccoyii Aquaculture 2006 254 40 45 10.1016/j.aquaculture.2005.10.013

[B15] Deveney MR Bayly TJ Johnston CJ Nowak BF A parasite survey of farmed Southern bluefin tuna, *Thunnus maccoyii *(Castelnau) J Fish Dis 2005 28 279 284 10.1111/j.1365-2761.2005.00629.x 15892753

[B16] Nowak BF Parasitic diseases in marine cage culture - An example of experimental evolution of parasites? Int J Parasitol 2007 37 581 588 10.1016/j.ijpara.2007.01.003 17316650

[B17] Whittington RJ Jones JB Hine PM Hyatt AD Epizootic mortality in the pilchard *Sardinops sagax neopilchardus *in Australia and New Zealand in 1995. 1. Pathology and epizootiology Dis Aquat Organ 1997 28 1 16

[B18] Ornamental fish http://www.ornamentalfish.org/aquanautmarket/petfishstats.php

[B19] Whittington RJ Chong R Global trade in ornamental fish from an Australian perspective: the case for revised import risk analysis and management strategies Prev Vet Med 2007 81 92 116 10.1016/j.prevetmed.2007.04.007 17485126

[B20] Lembo T Hampson K Haydon DT Craft M Dobson A Dushoff J Ernest E Hoare R Kaare M Mlengeya T Mentzel C Cleaveland S Exploring reservoir dynamics: a case study of rabies in the Serengeti ecosystem J Appl Ecol 2008 45 1246 1257 10.1111/j.1365-2664.2008.01468.x PMC3303133 22427710

[B21] Hedrick RP Movements of pathogens with the international trade of live fish: problems and solutions Rev Sci Tech 1996 15 523 531 8890378 10.20506/rst.15.2.938

[B22] Peeler EJ Oidtmann BC Midtlyng PM Miossec L Gozlan RE Non-native aquatic animals introductions have driven disease emergence in Europe Biol Invasions in press

[B23] Marcos-López M Gale P Oidtmann BC Peeler EJ Assessing the impact of climate change on disease emergence in freshwater fish in the United Kingdom Transbound Emerg Dis 2010 57 293 304 10.1111/j.1865-1682.2010.01150.x 20561287

[B24] Taylor NGH Dixon PF Jeffery KR Peeler EJ Denham KL Way K Koi herpesvirus: distribution and prospects for control in England and Wales J Fish Dis 2010 33 221 230 10.1111/j.1365-2761.2009.01111.x 19878413

[B25] Schikorski D Faury N Pepin JF Saulnier D Tourbiez D Renault T Experimental ostreid herpesvirus 1 infection of the Pacific oyster Crassostrea gigas: Kinetics of virus DNA detection by q-PCR in seawater and in oyster samples Virus Res 2011 155 28 34 10.1016/j.virusres.2010.07.031 20709119

[B26] Copp G Villizi L Gozlan R The demography of introduction pathways, propagule pressure and non-native freshwater fishes occurrence in England Aquatic Conserv: Mar Freshw Ecosyst 2010 20 595 601 10.1002/aqc.1129

[B27] Copp GH Templeton M Gozlan RE Propagule pressure and the invasion risks of non-native freshwater fishes: a case study in England J Fish Biol 2007 71 148 159

[B28] Smith P An overview of current fish disease research; Hyper-reality in a post-modern world? Bull Eur Assoc Fish Pathol 1999 19 310 312

[B29] Thorburn MA Factors influencing seasonal vibriosis mortality rates in Swedish pen reared rainbow trout Aquaculture 1987 67 79 85 10.1016/0044-8486(87)90010-X

[B30] Thorud K Djupvik H Infectious salmon anaemia in Atlantic salmon (*Salmo salar *L.) Bull Eur Assoc Fish Pathol 1988 8 109 111

[B31] Jarp J Gjevre AG Olsen AB Bruheim T Risk factors for furunculosis, infectious pancreatic necrosis and mortality in post-smolt Atlantic salmon, *Salmo salar *L J Fish Dis 1994 18 67 78

[B32] Jarp J Karlsen E Infectious salmon anaemia (ISA) risk factors in sea-cultured Atlantic salmon Salmo salar Dis Aquat Organ 1997 28 79 86

[B33] Jarp J Tangen K Willumsen FV Dyupvik HO Tveit AM Risk factors for infection with Aeromonas salmonicida subsp. salmonicida in Norwegian freshwater hatcheries Dis Aquat Organ 1993 17 81 86

[B34] Johnsen BO Jensen AJ The *Gyrodactylus *story in Norway Aquaculture 1991 98 289 302 10.1016/0044-8486(91)90393-L

[B35] Krkosek M Ford JS Morton A Lele S Myers RA Lewis MA Declining wild salmon populations in relation to parasites from farm salmon Science 2007 318 1772 1775 10.1126/science.1148744 18079401

[B36] Walker PJ Mohan CV Viral disease emergence in shrimp aquaculture: origins, impacts and the effectiveness of health management strategies Rev Aquac 2009 1 125 154 10.1111/j.1753-5131.2009.01007.x PMC7169130 32328167

[B37] Verner-Jeffreys DW Pond MJ Peeler EJ Rimmer GSE Oidtmann B Way K Mewett J Jeffrey K Bateman K Reese RA Feist SW Emergence of cold water strawberry disease of rainbow trout Oncorynchus mykiss in England and Wales: Outbreak investigations and transmission studies Dis Aquat Organ 2008 79 207 218 18589997 10.3354/dao01916

[B38] Segarra A Pépin JF Arzul I Morga B Faury N Renault T Detection and description of a particular Ostreid herpesvirus 1 genotype associated with massive mortality outbreaks of Pacific oysters, Crassostrea gigas, in France in 2008 Virus Res 2010 153 92 99 10.1016/j.virusres.2010.07.011 20638433

[B39] Haenen OLM Way K Bergmann SM Ariel E The emergence of koi herpesvirus and its significance to European aquaculture Bull Eur Ass Fish Pathol 2004 24 293 307

[B40] Daszak P Strieby A Cunningham AA Longcore JE Brown CC Porter D Experimental evidence that the bullfrog (Rana catesbeiana) is a potential carrier of chytridiomycosis, an emerging fungal disease of amphibians Herpetolog J 2004 14 201 207

[B41] Peeler EJ Murray AG Thebault A Brun E Giovaninni A Thrush MA The application of risk analysis in aquatic animal health management Prev Vet Med 2007 81 3 20 10.1016/j.prevetmed.2007.04.012 17544160

[B42] O.I.E Section 2.2. Risk analysis Aquatic Animal Health Code 2009 1 10 Paris: World Animal Health Organisation

[B43] WTO Agreement on the Application of Sanitary and Phytosanitary Measures Book Agreement on the Application of Sanitary and Phytosanitary Measures 1995 City: World Trade Organisation 21 10.20506/rst.15.2.945 8890392

[B44] Kahn SA Beers PT Findlay VL Peebles IR Durham PJ Wilson DW Gerrity SE Import Risk Analysis on Non-viable Salmonids and Non-salmonids Marine Finfish Book Import Risk Analysis on Non-viable Salmonids and Non-salmonids Marine Finfish 1999 City: Australian Quarantine and Inspection Service 409

[B45] Gaughan DJ Disease-translocation across geographic boundaries must be recognized as a risk even in the absence of disease identification: the case with Australian Sardinops Rev Fish Biol Fish 2002 11 113 123

[B46] Bridges VE Akkina J Grannis J Johnson C Johnson R Tuszynski C A qualitative assessment tool for the potential of infectious disease emergence and spread Prev Vet Med 2007 81 80 91 10.1016/j.prevetmed.2007.04.008 17498827

[B47] Woolhouse MEJ Population biology of emerging and re-emerging pathogens Trends Microbiol 2002 10 3 7 10.1016/S0966-842X(02)02428-9 12377561

[B48] Woolhouse MEJ Where do emerging pathogens come from? Microbe 2006 1 511 515

[B49] Woolhouse MEJ Epidemiology: emerging diseases go global Nature 2008 451 898 899 10.1038/451898a 18288175 PMC7095335

[B50] Brown SP Hochberg ME Grenfell BT Does multiple infection select for virulence? Trends Microbiol 2002 10 401 405 10.1016/S0966-842X(02)02413-7 12217504

[B51] Davies CM Webster JP Woolhouse MEJ Trade-offs in the evolution of virulence in an indirectly transmitted macroparasite Proc Biol Sci 2001 268 251 257 10.1098/rspb.2000.1367 11217894 PMC1088599

[B52] Frank SA Models of parasite virulence Q Rev Biol 1996 71 37 78 10.1086/419267 8919665

[B53] Haydon DT Cleaveland S Taylor LH Laurenson MK Identifying reservoirs of infection: A conceptual and practical challenge Emerg Infect Dis 2002 8 1468 1473 12498665 10.3201/eid0812.010317 PMC2738515

[B54] Woolhouse MEJ Gowtage-Sequeria S Host range and emerging and reemerging pathogens Emerg Infect Dis 2005 11 1842 1847 16485468 10.3201/eid1112.050997 PMC3367654

[B55] Woolhouse MEJ Haydon DT Antia R Emerging pathogens: the epidemiology and evolution of species jumps Trends Ecol Evol 2005 20 238 244 10.1016/j.tree.2005.02.009 16701375 PMC7119200

[B56] Woolhouse MEJ Taylor LH Haydon DT Population biology of multihost pathogens Science 2001 292 1109 1112 10.1126/science.1059026 11352066

[B57] Taylor LH Latham SM Woolhouse MEJ Risk factors for human disease emergence Philos Trans R Soc Lond B Biol Sci 2001 356 983 989 10.1098/rstb.2001.0888 11516376 PMC1088493

[B58] Woolhouse M Gaunt E Ecological origins of novel human pathogens Crit Rev Microbiol 2007 33 231 242 10.1080/10408410701647560 18033594

[B59] Maccrimmon HR World distribution of rainbow trout (*Salmon gairdneri*) J Fish Res Board Can 1971 28 663 704 10.1139/f71-098

[B60] Amos K Thomas J Disease interactions between wild and cultured fish: observation and lessons learned in the Pacific Northwest Bull Eur Ass Fish Pathol 2002 22 95 102

[B61] Coutant CC What is normative for fish pathogens? A perspective on the controversy over interactions between wild and cultured fish J Aquat Anim Health 1998 10 101 106 10.1577/1548-8667(1998)010<0101:WINFFP>2.0.CO;2

[B62] Mennerat A Nilsen F Ebert D Skorping A Intensive Farming: Evolutionary Implications for Parasites and Pathogens Evol Biol 2010 37 59 67 10.1007/s11692-010-9089-0 21151485 PMC2987527

[B63] Rogers DJ Randolph SE Studying the global distribution of infectious diseases using GIS and RS Nat Rev Microbiol 2003 1 231 237 10.1038/nrmicro776 15035027 PMC7096846

[B64] Bovo G Giorgetti G Jørgensen PEV Olesen NJ Infectious hematopoietic necrosis: first detection in Italy Bull Eur Ass Fish Pathol 1987 7 124

[B65] Enzmann PJ Kurath G Fichtner D Bergmann SM Infectious hematopoietic necrosis virus: monophyletic origin of European IHNV isolates from North-American genogroup M Dis Aquat Organ 2005 66 187:195 16261933 10.3354/dao066187

[B66] Basurco B Benmansour A Distant strains of the fish rhabdovirus VHSV maintain a sixth functional cistron which codes for a nonstructural protein of unknown function Virology 1995 212 741 745 10.1006/viro.1995.1534 7571446

[B67] Basurco B Vende P Monnier AF Winton JR de Kinkelin P Benmansour A Genetic diversity and phylogenetic classification of viral hemorrhagic septicemia virus (VHSV) Vet Res 1995 26 460 463 8581023

[B68] Cunningham CO Snow M Genetic analysis of infectious salmon anaemia virus (ISAV) from Scotland Dis Aquat Organ 2000 41 1 8 10907133 10.3354/dao041001

[B69] Kibenge FSB Lyaku JR Rainnie D Hammell KL Growth of infectious salmon anaemia virus in CHSE-214 cells and evidence for phenotypic differences between virus strains J Gen Virol 2000 81 143 150 10640552 10.1099/0022-1317-81-1-143

[B70] Kibenge FS Godoy MG Wang Y Kibenge MJ Gherardelli V Mansilla S Lisperger A Jarpa M Larroquete G Avendão F Lara M Gallardo A Infectious salmon anaemia virus (ISAV) isolated from the ISA disease outbreaks in Chile diverged from ISAV isolates from Norway around 1996 and was disseminated around 2005, based on surface glycoprotein gene sequences Virol J 2009 6 88 10.1186/1743-422X-6-88 19558648 PMC2710322

[B71] Godoy MG Aedo A Kibenge MJT Groman DB Yason CV Grothusen H Lisperguer A Calbucura M Avendaño F Imilán M Jarpa M Kibenge FS First detection, isolation and molecular characterization of infectious salmon anaemia virus associated with clinical disease in farmed Atlantic salmon (Salmo salar) in Chile BMC Vet Res 2008 4 28 10.1186/1746-6148-4-28 18680586 PMC2519066

[B72] Cottam EM Thébaud G Wadsworth J Gloster J Mansley L Paton DJ King DP Haydon DT Integrating genetic and epidemiological data to determine transmission pathways of foot-and-mouth disease virus Proc Biol Sci 2008 275 887 895 10.1098/rspb.2007.1442 18230598 PMC2599933

[B73] Salman MD Animal disease surveillance and survey systems - methods and applications 2003 1 Iowa: Iowa State Press

[B74] Cameron A Survey toolbox for aquatic animal diseases. A practical Manual and Software Package Book Survey toolbox for aquatic animal diseases. A practical Manual and Software Package 2002 City: ACIAR 375

[B75] East IJ Black PF McColl KA Hodgson RAJ Bernoth EM Survey for the presence of White Spot Syndrome virus in Australian crustaceans Aust Vet J 2004 82 236 240 10.1111/j.1751-0813.2004.tb12688.x 15149077

[B76] Lindenstroem T Nielsen B Buchmann K Gyrodactylids on salmonids from Danish streams Systematics and Phylogeny of Platyhelminthes; St Petesburg, Russia 1999 62 6421776891

[B77] Nielsen CV Buchmann K Occurrence of *Gyrodactylus *parasites in Danish fish farms Bull Eur Assoc Fish Pathol 2001 21 19 25

[B78] Wahli T Bernet D Steiner PA Schmidt-Posthaus H Geographic distribution of *Tetracapsuloides bryosalmonae *infected fish in Swiss rivers: an update Aquat Sci 2007 69 3 10 10.1007/s00027-006-0843-4

[B79] Wahli T Knuesel R Bernet D Segner H Pugovkin D Burkhardt-Holm P Escher M Schmidt-Posthaus H Proliferative kidney disease in Switzerland: current state of knowledge J Fish Dis 2002 25 491 500 10.1046/j.1365-2761.2002.00401.x

[B80] Feist SW Peeler EJ Gardiner R Smith E Longshaw M Proliferative kidney disease and renal myxosporidiosis in juvenile salmonids from rivers in England and Wales J Fish Dis 2002 25 451 458 10.1046/j.1365-2761.2002.00361.x

[B81] Peeler EJ Feist SW Longshaw M Thrush MA St-Hilaire S An assessment of the variation in the prevalence of renal myxosporidiosis and hepatitis in wild brown trout, *Salmo trutta *L., within and between rivers in South-West England J Fish Dis 2008 31 719 728 10.1111/j.1365-2761.2008.00942.x 18681903

[B82] Seagrave CP Bucke D Hudson EB McGregor D A survey of the prevalence and distribution of proliferative kidney disease (PKD) in England and Wales J Fish Dis 1981 4 437 439 10.1111/j.1365-2761.1981.tb01155.x

[B83] Chambers E Gardiner R Peeler EJ An investigation into the prevalence of *Renibacterium salmoninarum *in farmed rainbow trout (*Onchorhynchus mykiss*) and wild fish populatins in selected river catchments in England and Wales between 1998 and 2000 J Fish Dis 2008 31 89 96 10.1111/j.1365-2761.2007.00868.x 18234016

[B84] Austin B Rayment JN Epizootiology of Renibacterium salmoninarum, the causal agent of bacterial kidney disease in salmonid fish J Fish Dis 1985 8 505 509 10.1111/j.1365-2761.1985.tb00965.x

[B85] Rodger HD Turnbull T Muir F Millar S Richards RH Infectious salmon anemia (ISA) in the United Kingdom Bull Eur Assoc Fish Pathol 1998 18 115 116

[B86] Raynard RS Murray AG Gregory A Infectious salmon anaemia virus in wild fish from Scotland Dis Aquat Organ 2001 46 93 100 11678233 10.3354/dao046093

[B87] Martin PAJ Current value of historical and ongoing surveillance for disease freedom: Surveillance for bovine Johne's disease in Western Australia Prev Vet Med 2008 84 291 309 10.1016/j.prevetmed.2007.12.002 18243373

[B88] Martin PAJ Cameron AR Barfod K Sergeant ESG Greiner M Demonstrating freedom from disease using multiple complex data sources. 2: Case study-Classical swine fever in Denmark Prev Vet Med 2007 79 98 115 10.1016/j.prevetmed.2006.09.007 17239459

[B89] Weinstock MA A generalised scan statistic test for the detection of clusters Int J Epidemiol 1981 10 289 293 10.1093/ije/10.3.289 7287289

[B90] Clark PJ Evans FC Distance to nearest neighbor as a measure of special relationship in populations Ecology 1954 159 163

[B91] Carpenter TE Methods to investigate spatial and temporal clustering in veterinary epidemiology Prev Vet Med 2001 48 303 320 10.1016/S0167-5877(00)00199-9 11259822

[B92] Ward MP Carpenter TE Techniques for analysis of disease clustering in space and in time in veterinary epidemiology Prev Vet Med 2000 45 257 284 10.1016/S0167-5877(00)00133-1 10821965

[B93] Heuch PA Revie CW Gettinby G A comparison of epidemiological patterns of salmon lice, Lepeophtheirus salmonis, infections on farmed Atlantic salmon, Salmo salar L., in Norway and Scotland J Fish Dis 2003 26 539 551 10.1046/j.1365-2761.2003.00490.x 14575372

[B94] Revie CW Hollinger E Gettinby G Lees F Heuch PA Clustering of parasites within cages on Scottish and Norwegian salmon farms: Alternative sampling strategies illustrated using simulation Prev Vet Med 2007 81 135 147 10.1016/j.prevetmed.2007.04.004 17532070

[B95] Lees F Gettinby G Revie CW Changes in epidemiological patterns of sea lice infestation on farmed Atlantic salmon, Salmo salar L., in Scotland between 1996 and 2006 J Fish Dis 2008 31 259 268 10.1111/j.1365-2761.2007.00897.x 18353017

[B96] Longshaw M Frear PA Nunn AD Cowx IG Feist SW The influence of parasitism on fish population success Fish Manag Ecol 2010 17 426 434 10.1111/j.1365-2400.2010.00741.x

[B97] Georgiadis MP Johnson WO Gardner IA Sample size determination for estimation of the accuracy of two conditionally independent tests in the absence of a gold standard Prev Vet Med 2005 71 1 10 10.1016/j.prevetmed.2005.04.004 16076507

[B98] Georgiadis MP Johnson WO Gardner IA Singh R Correlation-adjusted estimation of sensitivity and specificity of two diagnostic tests Appl Stat 2003 52 63 76

[B99] Nérette P Stryhn H Dohoo I Hammell L Using pseudogold standards and latent-class analysis in combination to evaluate the accuracy of three diagnostic tests Prev Vet Med 2008 85 207 225 10.1016/j.prevetmed.2008.01.011 18355935

[B100] Nérette P Dohoo I Hammell L Estimation of specificity and sensitivity of three diagnostic tests for infectious salmon anaemia virus in the absence of a gold standard J Fish Dis 2005 28 89 99 10.1111/j.1365-2761.2005.00612.x 15705154

[B101] O.I.E Manual of Diagnostic Tests for Aquatic Animals 2009 5 Paris: Office International des Epizooties

[B102] Williams CJ Moffitt CM Estimation of fish and wildlife disease prevalence from imperfect diagnostic tests on pooled samples with varying pool sizes Ecol Inform 2010 5 273 280 10.1016/j.ecoinf.2010.04.003

[B103] Keeling MJ Woolhouse MEJ May RM Davies G Grenfell BT Modelling vaccination strategies against foot-and-mouth disease Nature 2003 421 136 142 10.1038/nature01343 12508120

[B104] Ortiz-Pelaez A Pfeiffer DU Soares-Magalhaes RJ Guitian FJ Use of social network analysis to characterize the pattern of animal movements in the initial phases of the 2001 foot and mouth disease (FMD) epidemic in the UK Prev Vet Med 2006 76 40 55 10.1016/j.prevetmed.2006.04.007 16769142

[B105] Picado A Guitian FJ Pfeiffer DU Space-time interaction as an indicator of local spread during the 2001 FMD outbreak in the UK Prev Vet Med 2007 79 3 19 10.1016/j.prevetmed.2006.11.009 17175049

[B106] Paisley LG Karlsen E Jarp J Mo TA A Monte Carlo simulation model for assessing the risk of introduction of *Gyrodactylus salaris *to the Tana river, Norway Dis Aquat Organ 1999 37 145 152 10494504 10.3354/dao037145

[B107] Høgåsen HR Brun E Risk of inter-river transmission of *Gyrodactylus salaris *by migrating Atlantic salmon smolts, estimated by Monte Carlo simulation Dis Aquat Organ 2003 57 247 254 14960038 10.3354/dao057247

[B108] Peeler EJ Gardiner R Thrush MA Qualitative risk assessment of routes of transmission of the exotic fish parasite *Gyrodactylus salaris *between river catchments in England and Wales Prev Vet Med 2004 64 175 189 10.1016/j.prevetmed.2004.05.005 15325771

[B109] Anderson RM May RM Population biology of infectious diseases: Part 1 Nature 1979 280 361 367 10.1038/280361a0 460412

[B110] Murray AG A model of the emergence of infectious pancreatic necrosis virus in Scottish salmon farms 1996-2003 Ecol Modell 2006 199 64 72 10.1016/j.ecolmodel.2006.06.010

[B111] Ruane NM Murray AG Geoghegan F Raynard RS Modelling the spread of Infectious Pancreatic Necrosis Virus (IPNV) in the Irish salmon farming industry: the role of inputs Ecol Modell 2009 220 1369 1374 10.1016/j.ecolmodel.2009.02.016

[B112] Taylor NGH Norman RA Way K Peeler EJ Modelling the koi herpesvirus (KHV) epidemic highlights the importance of active surveillance within a national control policy J Appl Ecol 2011 48 348 355 10.1111/j.1365-2664.2010.01926.x

[B113] Thrush M Peeler E Stochastic simulation of live salmonid movement in England and Wales to predict potential spread of exotic pathogens Dis Aquat Organ 2006 72 115 123 17140134 10.3354/dao072115

[B114] Green DM A strategic model for epidemic control in aquaculture Prev Vet Med 2010 94 119 127 10.1016/j.prevetmed.2009.12.004 20106539

[B115] Green DM Gregory A Munro LA Small- and large-scale network structure of live fish movements in Scotland Prev Vet Med 2009 91 261 269 10.1016/j.prevetmed.2009.05.031 19625093

[B116] Sharkey KJ Fernandez C Morgan KL Peeler E Thrush M Turnbull JF Bowers RG Pair-level approximations to the spatio-temporal dynamics of epidemics on asymmetric contact networks J Math Biol 2006 53 61 85 10.1007/s00285-006-0377-3 16791650

[B117] Murray AG Gillibrand PA Modelling dispersal of salmon lice in Loch Torridon, Scotland Mar Pollut Bull 2006 53 128 135 10.1016/j.marpolbul.2005.09.013 16246378

[B118] Murray AG Amundrud TL Gillibrand PA Models of hydrodynamic pathogen dispersal affecting Scottish salmon production: modelling shows how Scotland eradicated ISA, but not IPN Bull Aquacult Assoc Can 2005 105 79 86

[B119] Viljugrein H Staalstrøm A Molvær J Urke H Jansen P Integration of hydrodynamics into a statistical model on the spread of pancreas disease (PD) in salmon farming Dis Aquat Organ 2009 88 35 44 20183963 10.3354/dao02151

[B120] Murray AG Amundrud TL Gillibrand PA Models of hydrodynamic pathogen dispersal affecting Scottish salmon production: modelling shows how Scotland eradicated ISA, but not IPN Bull Aquacult Assoc Can 2005 105 80 87

[B121] Keeling MJ Woolhouse MEJ Shaw DJ Mattthews L Chase-Topping M Haydon DT Cornell SJ Kappey J Wilesmith JW Grenfell BT Dynamics of the 2001 foot and mouth epidemic: stochastic dispersal and a hetergenous landscape Science 2001 294 812 817 10.1126/science.1064627 11679661

[B122] Jansen PA Matthews L Toft N Geographic risk factors for inter-river dispersal of Gyrodactylus salaris in fjord-systems in Norway Dis Aquat Organ 2007 74 139 149 17432043 10.3354/dao074139

[B123] Vågsholm I Djupvik HO Willumsen FV Tveit AM Tangen K Infectious salmon anaemia (ISA) epidemiology in Norway Prev Vet Med 1994 19 277 290 10.1016/0167-5877(94)90095-7

[B124] Murray AG Smith RJ Stagg RM Shipping and the spread of infectious salmon anemia in Scottish aquaculture Emerg Infect Dis 2002 8 1 5 10.3201/eid0801.010144 11749740 PMC2730283

[B125] McClure CA Hammell KL Dohoo IR Risk factors for outbreaks of infectious salmon anemia in farmed Atlantic salmon, Salmo salar Prev Vet Med 2005 72 263 280 10.1016/j.prevetmed.2005.07.010 16188335

[B126] Taylor NGH Way K Jeffery KR Peeler EJ The role of live fish movements in spreading koi herpesvirus throughout England and Wales J Fish Dis 2010 33 1005 1007 10.1111/j.1365-2761.2010.01198.x 21155075

[B127] Revie CW Gettinby G Treasurer JW Wallace C Identifying epidemiological factors affecting sea lice *Lepeophtheirus salmonis *abundance on Scottish salmon farms using general linear models Dis Aquat Organ 2003 57 85 89 14735925 10.3354/dao057085

[B128] Corsin F Turnbull JF Hao NV Mohan CV Phi TT Phuoc LH Tinh NTN Morgan KL Risk factors associated with white spot syndrome virus infection in a Vietnamese rice-shrimp farming system Dis Aquat Organ 2001 47 1 12 11797910 10.3354/dao047001

[B129] St-Hilaire S Ellis T Cooke A North BP Turnbull JF Knowles T Kestin S Fin erosion on rainbow trout on commercial trout farms in the United Kingdom Vet Rec 2006 159 446 451 10.1136/vr.159.14.446 17012609

[B130] Ersdal C Midtlyng PJ Jarp J An epidemiological study of cataracts in seawater farmed Atlantic salmon Salmo salar Dis Aquat Organ 2001 45 229 236 11558732 10.3354/dao045229

[B131] Vaagsholm I Djupvik HO Risk factors for skin lesions in Atlantic salmon, Salmo salar L J Fish Dis 1998 21 449 453 10.1046/j.1365-2761.1998.00123.x 29739172

[B132] Thrush MA Murray AG Brun E Wallace S Peeler EJ The application of risk and disease modelling to emerging freshwater diseases in wild aquatic animals Freshwat Biol 2011 56 658 675 10.1111/j.1365-2427.2010.02549.x

[B133] Taylor NGH Sommerville C Wootten R The epidemiology of Argulus spp. (Crustacea: Branchiura) infections in stillwater trout fisheries J Fish Dis 2006 29 193 200 10.1111/j.1365-2761.2006.00704.x 16635059

[B134] Thorburn MA Variables associated with the use of chemotherapeutic agents on trout farms in Ontario, Canada Prev Vet Med 1995 24 1 14 10.1016/0167-5877(95)00472-9 11448503

[B135] Vågsholm I Djupvik HO Risk factors for abdominal adhesions in Atlantic salmon, *Salmo salar *L J Fish Dis 1999 22 53 58 10.1046/j.1365-2761.1999.00137.x 29739172

[B136] Vågsholm I Djupvik HO Risk factors for spinal deformities in Atlantic salmon, *Salmo salar *L J Fish Dis 1998 21 47 53 10.1046/j.1365-2761.1998.00069.x 29739172

[B137] Wheatley SB McLoughlin MF Menzies FD Goodall EA Site management factors influencing mortality rates in Atlantic salmon (*Salmo salar *L.) during marine production Aquaculture 1995 136 195 207 10.1016/0044-8486(95)01058-0

[B138] Aunsmo A Valle PS Sandberg M Midtlyng PJ Bruheim T Stochastic modelling of direct costs of pancreas disease (PD) in Norwegian farmed Atlantic salmon (Salmo salar L.) Prev Vet Med 2010 93 233 241 10.1016/j.prevetmed.2009.10.001 19931201

[B139] Revie CW Gettinby G Treasurer JW Rae GH Clark N Temporal, environmental and management factors influencing the epidemiological patterns of sea lice (*Lepeophtheirus salmonis*) infestations on farmed Atlantic salmon (*Salmo salar*) in Scotland Pest Manag Sci 2002 58 576 584 10.1002/ps.476 12138624

[B140] Revie CW Gettinby G Treasurer JW Rae GH The epidemiology of the sea lice, *Caligus elongatus *Nordmann, in marine aquaculture of Atlantic salmon, *Salmo salar *L. in Scotland J Fish Dis 2002 25 391 400 10.1046/j.1365-2761.2002.00388.x

[B141] Zagmutt-Vergara FJ Carpenter TE Farver TB Hedrick RP Spatial and temporal variations in sea lice (Copeda: Caligidae) infestations of three salmonid species farmed in net pens in southern Chile Dis Aquat Organ 2005 64 163 173 15918480 10.3354/dao064163

[B142] McKenzie E Gettinby G McCart K Revie CW Time-series models of sea lice Caligus elongatus (Nordmann) abundance on Atlantic salmon Salmo salar L. in Loch Sunart, Scotland Aquacult Res 2004 35 764 772 10.1111/j.1365-2109.2004.01099.x

[B143] Tucker CS Norman R Shinn AP Bron JE Sommerville C Wooten R A single cohort time delay model of the life-cycle of the salmon louse *Lepeophtheirus salmonis *on Atlantic salmon *Salmo salar* Fish Pathol 2002 37 107 118 10.3147/jsfp.37.107

[B144] Pike AW Wadsworth SL Sea lice on salmonids: their biology and control Adv Parasitol 2000 44 231 337 10.1016/s0065-308x(08)60233-x 10563397

[B145] Johnsen BO Jensen AJ Infestations of Atlantic salmon, *Salmo salar*, by *Gyrodactylus salaris *in Norwegian rivers J Fish Biol 1986 29 233 241 10.1111/j.1095-8649.1986.tb04941.x

[B146] Taylor NGH Wootten R Sommerville C The influence of risk factors on the abundance, egg laying habits and impact of Argulus foliaceus in stillwater trout fisheries J Fish Dis 2009 32 509 519 10.1111/j.1365-2761.2009.01007.x 19460088

[B147] Murphy PJ St-Hilaire S Bruer S Corn PS Peterson CR Distribution and pathogenicity of Batrachochytrium dendrobatidis in boreal toads from the Grand Teton area of western Wyoming Ecohealth 2009 6 109 120 10.1007/s10393-009-0230-4 19418097

[B148] Patterson KR Modelling the impact of disease-induced mortality in an exploited population of the fungal parasite Icthyophonus hoferi in the North Sea herring (*Clupea harengus*) Can J Fish Aquat Sci 1996 53 2870 2877

[B149] Borsuk ME Reichert P Peter A Schager E Burkhardt-Holm P Assessing the decline of brown trout (*Salmo trutta*) in Swiss rivers using a Bayesian probability network Ecol Model 2006 192 224 244 10.1016/j.ecolmodel.2005.07.006

[B150] Murray AG O'Callaghan M The numerical and programming methods used to implement models of the spread and impact of a major epidemic disease: pilchard herpesvirus, Australia 1995 and 1998/1999 Environ Model Software 2005 20 575 585 10.1016/j.envsoft.2004.03.011

[B151] Barber I Scharsack JP The three-spined stickleback-*Schistocephalus solidus *system: an experimental model for investigating host-parasite interactions in fish Parasitology 2010 137 411 424 10.1017/S0031182009991466 19835650

[B152] Georgiadis MP Gardner IA Hedrick RP The role of epidemiology in the prevention, diagnosis and control of infectious disease of fish Prev Vet Med 2001 48 287 302 10.1016/S0167-5877(00)00202-6 11259821

